# Authors’ Reply: Response to “Older Cancer Patients’ User Experiences With Web-Based Health Information Tools: A Think-Aloud Study”

**DOI:** 10.2196/jmir.6934

**Published:** 2016-11-16

**Authors:** Sifra Bolle, Geke Romijn, Ellen M A Smets, Eugene F Loos, Marleen Kunneman, Julia C M van Weert

**Affiliations:** ^1^Department of Communication ScienceAmsterdam School of Communication Research (ASCoR)University of AmsterdamAmsterdamNetherlands; ^2^Department of Clinical PsychologyFaculty of Behavioural and Movement SciencesVU UniversityAmsterdamNetherlands; ^3^Department of Medical PsychologyAmsterdam Medical Center (AMC)University of AmsterdamAmsterdamNetherlands; ^4^Department of Medical Decision MakingLeiden University Medical CenterLeidenNetherlands

We greatly appreciate the thoughtful comments of Gokani and colleagues [1] in response to our article “Older Cancer Patients’ User Experiences With Web-Based Health Information Tools: A Think-Aloud Study” [2]. We are happy to elaborate on the points for which they request further clarification. 

First, they have concerns about our recruitment strategy of study participants via a patient panel (PanelCom) that would lead to participants being more experienced Internet users as compared to the average older adult. However, it is a misunderstanding that PanelCom is a service which “recruits cancer patients via email.” As explained in the paper (under the subheading ‘study design, setting, and sample’ in the methods section), PanelCom is a panel of cancer patients who previously participated in studies of the Departments of Communication Science and Medical Psychology and consented to be contacted again in future studies. These previous studies were not necessarily online; especially older participants were mostly recruited in hospitals. Nevertheless, (older) patients that have no experience with Web-based technology are not likely to use Web-based health information tools. Hence they were not the target population of this study. However, 61% of our sample does consist of participants that have no to very little experience in using a computer or tablet (ie, 0-2 hours per week; see table 1 in the paper).

The second point of concern that Gokani et al raise is that the usage pattern of the websites we have tested might be different had we also taken search queries in search engines such as Google into account. We agree (under subheading ‘cancer information websites’ under materials in the methods section, where we mention that people tend to look no further than the first page of the search results), and we took this into account by selecting two websites that were the first results on Google for searches for the Dutch words for “chemotherapy,” “cancer,” and “hospital.” Furthermore, the aim of the current study was to identify usability issues in order to make recommendations for the design of usable Web-based health information tools for older patients as a preparation for the systematic development of a web-based health information tool, the Patient Navigator. The Patient Navigator will be provided by hospitals and healthcare providers. This means that users will directly access the website rather than a search engine. The question how older (cancer) patients search for online health information covering the whole navigational usage pattern remains an interesting question for future research. Moreover, we agree that the factors suggested by Gokani et al such as currency, authorship, and bias contained within healthcare might influence perceived usefulness and that these factors should be investigated in future research. 

Third, Gokani et al suggest that recommendations are needed that enhance the ability to personalize Web-based tools rather than generalized recommendation. Indeed, the digital nature of Web-based tools allows the tailoring of the design and information to individual needs and preferences of patients, which is why we recommend tailoring on websites for older cancer patients (under the subheading ‘comparison with prior work and practical implications’ in the discussion section). A simple way of self-tailoring is our recommendation to “avoid large amounts of information on a page. If possible, display options on 1 page, for example, first provide an overview with options, and then (after visitors choose what information they wish to read) the relevant information.” Limited information on a webpage would make it possible to provide patients with a large font size to enable them to avoid scrolling. We agree with Gokani et al that more research is needed on other ways of tailoring that could benefit older patients, such as mode tailoring and message frame tailoring, next to content tailoring (see work by our research group [3,4]).

Finally, Gokani et al comment that “the true value of integrating the tools within the patient consultation could also be further explored.”  We couldn’t agree more with this comment. Hence, this is the next step in our research project. We expect that the Patient Navigator will help patients in processing information and in preparing for the consultation with their healthcare provider. At the moment we are collecting the data to evaluate the clinical use of the Patient Navigator.
